# Non-gelated polymeric photonic crystal films

**DOI:** 10.3389/fchem.2022.1009669

**Published:** 2022-09-20

**Authors:** Zhaoran Chu, Zheng Ding, Xuanjun Ning, Yimihan A, Menghan Wang, Kan Shao, Wenwei Tang, Cheng Chen, Jianzhong Bai

**Affiliations:** ^1^ School of Energy and Materials, Shanghai Polytechnic University, Shanghai, China; ^2^ Department of Orthopaedics, Tongren Hospital, Shanghai Jiao Tong University School of Medicine, Shanghai, China; ^3^ Department of Endocrinology, Tongren Hospital, Shanghai Jiao Tong University School of Medicine, Shanghai, China; ^4^ Modern Service Department, College of International Vocational Education, Shanghai Polytechnic University, Shanghai, China; ^5^ Shanghai Engineering Research Center of Advanced Thermal Functional Materials, Shanghai Polytechnic University, Shanghai, China

**Keywords:** polymer, bulk film, sensor, colloidal crystal, photonic crystal

## Abstract

A rapid curing method for the preparation of colloidal photonic crystal films is presented. Firstly, a colloidal crystal array template was prepared by self-assembly of nanospheres, and then a dilute polymer solution was poured into the gap of the template. Then the composite photonic film was obtained as the polymer solution was cured. Such films have good properties in mechanical strength, anti pH interference, rapid solvent response and are easy to preserve. The films show good linear response to ethanol aqueous solutions of different concentrations, and the response equilibrium takes less than 20 s. The films also show long-term stability and reusability, and further functionalization can make the films multi-sensitive.

## Introduction

The preparation of high-efficiency, low-cost chemical sensors is facing increasing demand in many different fields (Khanyile, 2022). In particular, the determining of ethanol concentration is crucial for industrial production fields including food, cosmetics and pharmaceuticals. Strict regulations on alcoholic beverages, and accurate ethanol measurement is essential. Despite the well-developed detection methods such as high-performance liquid chromatography, gas chromatography, near-infrared spectroscopy, and quantitative nuclear magnetic resonance, which have high sensitivity, but are expensive for small companies and can not realize on-site and real-time detection (Castellari et al., 2001). Moreover, the accuracy of alcohol measurement largely depends on the professional skills of operators. Hence, there is an urgent need for cheap chemical sensors that are easy to operate and distinguish, reliable and long-term stable.

Stimuli-responsive polymers have interesting characteristics such as shape memory, rehydratablity and volume phase transition, as the external environment changes slightly, the polymers have noticeable and reversible changes (Wang et al., 2021). Precisely, the combination of stimulus response hydrogels and photonic crystals (PC) can convert volume changes of polymers under external stimulation into color changes of PCs (Huang et al., 2014; Chen et al., 2018a), thus creating colorimetric sensors (Wang et al., 2022a; He et al., 2022). These colorimetric sensors have attracted more and more attention due to their simple detection and intuitive readout. PCs are uniform arrays of light scatterers that diffract electromagnetic wave with certain frequencies according to Bragg’s law (Liu et al., 2017), which can be produced by “top-down” and “bottom-up” strategies. Self-assembly of nanospheres is a convenient method to construct colloidal photonic crystal (CPC) that can effectively diffracts visible light. For instance, a vapor sensor constructed by PC and copolymer can adsorb alcohol vapor by relying on favorable solubility parameters, which increases the effective refractive index of the PC system. Therefore, as the PC sensor is exposed to volatile alcohol, a significant color change occurs, and the concentration of the alcohol vapor can be determined by the red-shift of the diffraction peak (Fu et al., 2022).

Poly (vinyl alcohol) (PVA) is a non-toxic, biodegradable polymer with good mechanical properties, which has certain resistance to organic solvents and film-forming ability (Chen et al., 2019). PVA-based materials have been used in biomedical fields, such as drug delivery, wound healing, ultrafiltration and tissue engineering scaffolds (De Lima et al., 2020; Wang et al., 2021). PVA/PC composite materials have been developed based on PVA hydrogels prepared by utilizing the gelation behavior of PVA aqueous solution under freezing conditions. The PVA/PC materials can be prepared in large quantities and are also suitable for chemical modification (Chen et al., 2010). On this basis, a series of PVA/PC sensors were developed for different sensing motifs and mechanisms including glucose sensing lens (Chen et al., 2017; Ruan et al., 2017; Tang and Chen, 2020) and films (Chen et al., 2018b), drug delivering lens (Chu et al., 2022) and metal ion sensing (Wang et al., 2022b).

In the current study, we facilely prepared an easy-to-use PVA/PC material based on the characteristics of PVA bulk film. Specifically, a CPC template was prepared by self-assembly of monodisperse nanospheres, and the template was then infiltrated with the preferred PVA solution. The composite films were formed by curing the system via simple thermal treatment. Compared with traditional hydrogel-based PC materials, the current test-paper-like films have good mechanical properties (Liu et al., 2017). Such films can be used as a colorimetric sensor to detect the ethanol concentration quickly. Further modification can make such films meet different sensing requirements.

## Materials and methods

### Materials

Styrene (St, 99%) and hydrogen peroxide solution (H_2_O_2_, 30%) were purchased from Shanghai Adamas Reagent Ltd. Methacrylic acid (MAA, 98%), ammonium persulfate (APS, 98.5%), sodium hydroxide (NaOH, 96%), PVA (1750 ± 50, alcoholysis degree 99%), ethanol (99.7%) were purchased from Sinopharm Chemical Reagent Co., Ltd. Milli-Q water (18.2 MΩ cm) was used throughout the experiment.

### Synthesis of monodisperse nanospheres

Five millilitre of MAA and 200 ml of water were added into a flask, the liquid was stirred and heated to boiling (Chen et al., 2015), then 80 ml of St (washed with 5% NaOH aqueous solution to remove impurities before use) monomer was added, and the mixture was heated to boiling again. Then, 5 ml water with 0.48 g APS was added to the mixture to initiate the reaction. The system was kept boiling for 30–60 min and the polymerization was finished by ice water baths. The resulting latex was centrifuged and transferred into a dialysis bag for dialysis against ultrapure water to remove small molecules.

### Self-assembly of colloidal photonic crystal template

One millilitre of dialyzed monodisperse PS nanospheres suspension was diluted into 100 ml with water, and then the glass slides (soaked in H_2_O_2_ before use) were vertically placed in the diluted PS suspension. The suspension was dried in an oven at 60°C for 48 h, the PS nanospheres were self-assembled onto the glass slides with the evaporation of water due to the capillary force, and the resulted CPC showed bright structural colors.

### Preparation of Poly vinyl alcohol/colloidal photonic crystal films

**FIGURE 1:**
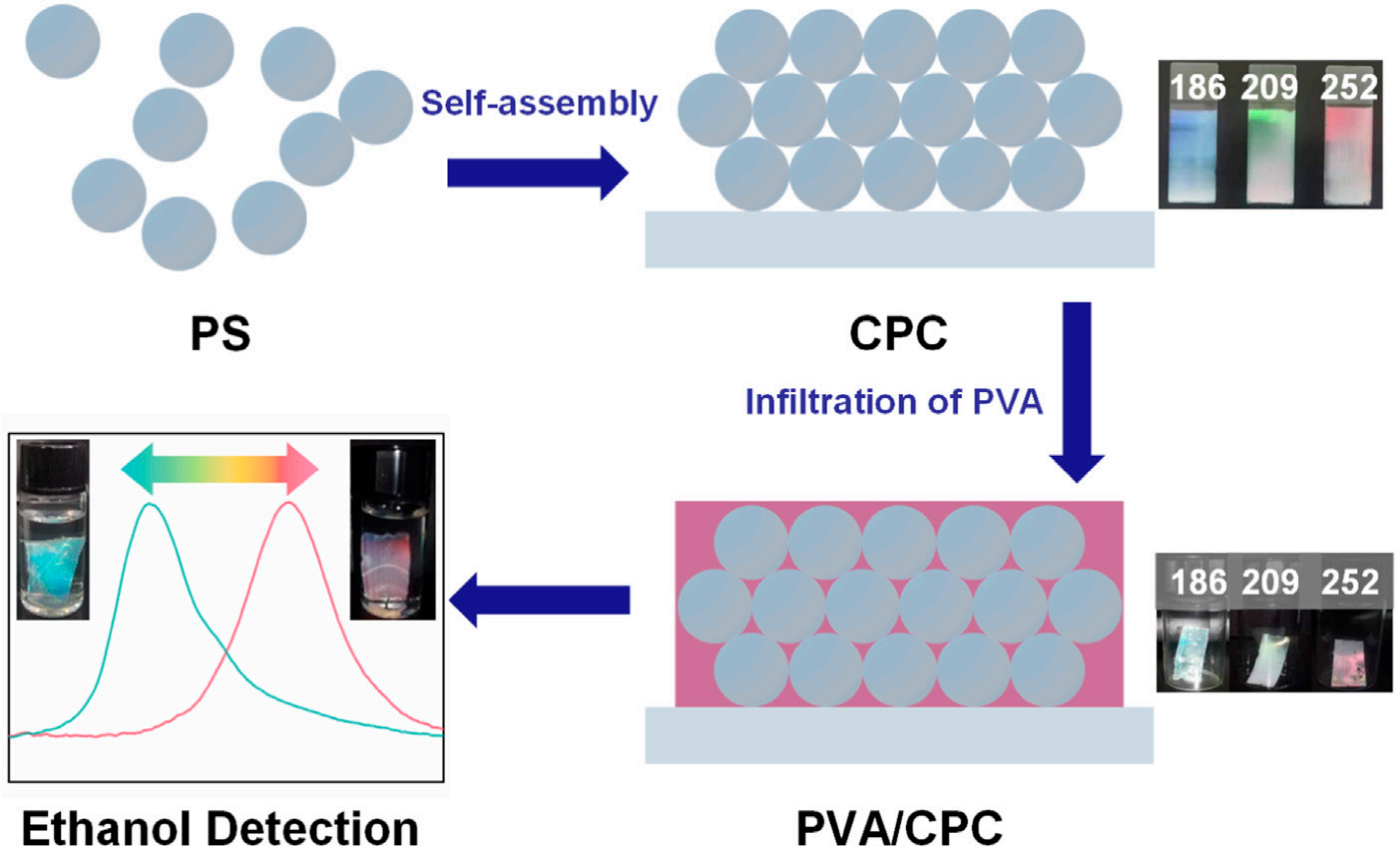
Construction illustration of PVA/CPC film sensor.

PVA powder was placed in water and kept stirring at 100°C for 2 h to form a ∼5% homogeneous transparent solution. The freshly prepared PVA solution was cooled to temperature, and meanwhile the air bubbles were eliminated. The PVA solution was then poured onto the slightly tilted CPC-covered glass slide, and the PVA solution flowed slowly down the glass slide while penetrating into the gap between the nanospheres of the CPC. The slide containing PVA infiltrated CPC was put into an oven to cure into a composite film at 60°C. After 2 h, the cured PVA/CPC film could be easily peeled off from the glass slide without damage, and the film was cut into the required size (2 cm × 1 cm) and stored without further treatment. The construction of the whole PVA/CPC film is shown in Figure 1. The detailed preparation results were shown in supporting information.

### Characterizations

Particle sizes and size distribution of PS nanospheres were analyzed by Zetasizer (Malvern, 3000HS). The morphology of the CPC template was characterized by a scanning electron microscope (SEM, Hitachi, S-4800). The optical photos of the samples were taken by a digital camera (Canon, EOS 6D) with a macro lens (Tamron, 272 E). The diffraction spectrum of the sample was captured by a fiber-optical spectrometer (Ocean Optics, USB 4000-XR1-ES) with a light source (Ocean Optics, DH-2000-BAL). The diffraction spectrum was collected with wavelength ranges between 400 and 900 nm.

In order to determine the ethanol sensitivity of PVA/CPC film, the film was immersed in ethanol aqueous solutions of different concentrations, and whose diffraction spectrum was measured as the swelling equilibrium of the film was reached. The equilibrium swelling degree (*S*) can be calculated as:

$$S=\frac{w_{1}-w_{0}}{w_{0}}\times 100\%$$ (1)

where *w*_0_ is the weight of the dried PVA/CPC film, and *w*_1_ is the weight of the swollen PVA/CPC film. The film was immersed in 20 ml of ethanol aqueous solution with different concentrations for 30 min to reach swelling equilibrium, and the excess water was wiped from the surface of the PVA/CPC film with tissue paper.

To investigate the mechanical properties of PVA/CPC film, the elongation was tested by fixing the fully swollen PVA/CPC film on the ruler with a dovetail clamp, and the film was slowly and uniformly stretched till the film broke. During the stretching process, the diffraction wavelength of the PVA/CPC film was recorded and the elongation (*E*) can be calculated as:

$$E=\frac{l_{1}-l_{0}}{l_{0}}\times 100\%$$ (2)

where l0 is the initial length of PVA/CPC film while l1 is the length of elongated PVA/CPC film.

For biocompatibility investigation, cell culture was conducted (Tang and Chen, 2020). Inverted fluorescence microscopy (IFM) observation was employed to evaluate the viability and morphology after adding Calcein acetoxymethyl (AM) and propidium iodide (PI) for the staining of living cells and dead cells in 24 and 48 h, respectively.

## Results and discussions

The critical design basis of this study is the characteristics of different PVA materials. According to the alcoholysis degree, PVA is usually divided into high hydrolyzed (>98%) and low hydrolyzed (<88%); on the other hand, according to the polymerization degree, usually from 300 to 6,000, the molecular weight difference in PVA molecule is rather huge. PVA with low alcoholysis degree can be dissolved at room temperature with low viscosity, which cannot form hydrogel via physical method due to the low hydroxyl content. PVA with high alcoholysis degree and medium molecular weight cannot be dissolved in water at room temperature, but only swells. Highly hydrolyzed PVA must be dissolved in water at high temperature and the related hydrogels can be formed through the freezing method, relying on enough hydrogen bonds. Hence, in the current study, a highly hydrolyzed, medium molecular weight PVA was chosen to fabricate bulk films combined with CPC. The as-prepared PVA/CPC films are insoluble but swellable in water at room temperature, which have higher mechanical strength and is easier to preserve than PVA hydrogel-based photonic crystal materials, and also have swelling sensing properties and funtionalization potentials.

**FIGURE 2:**
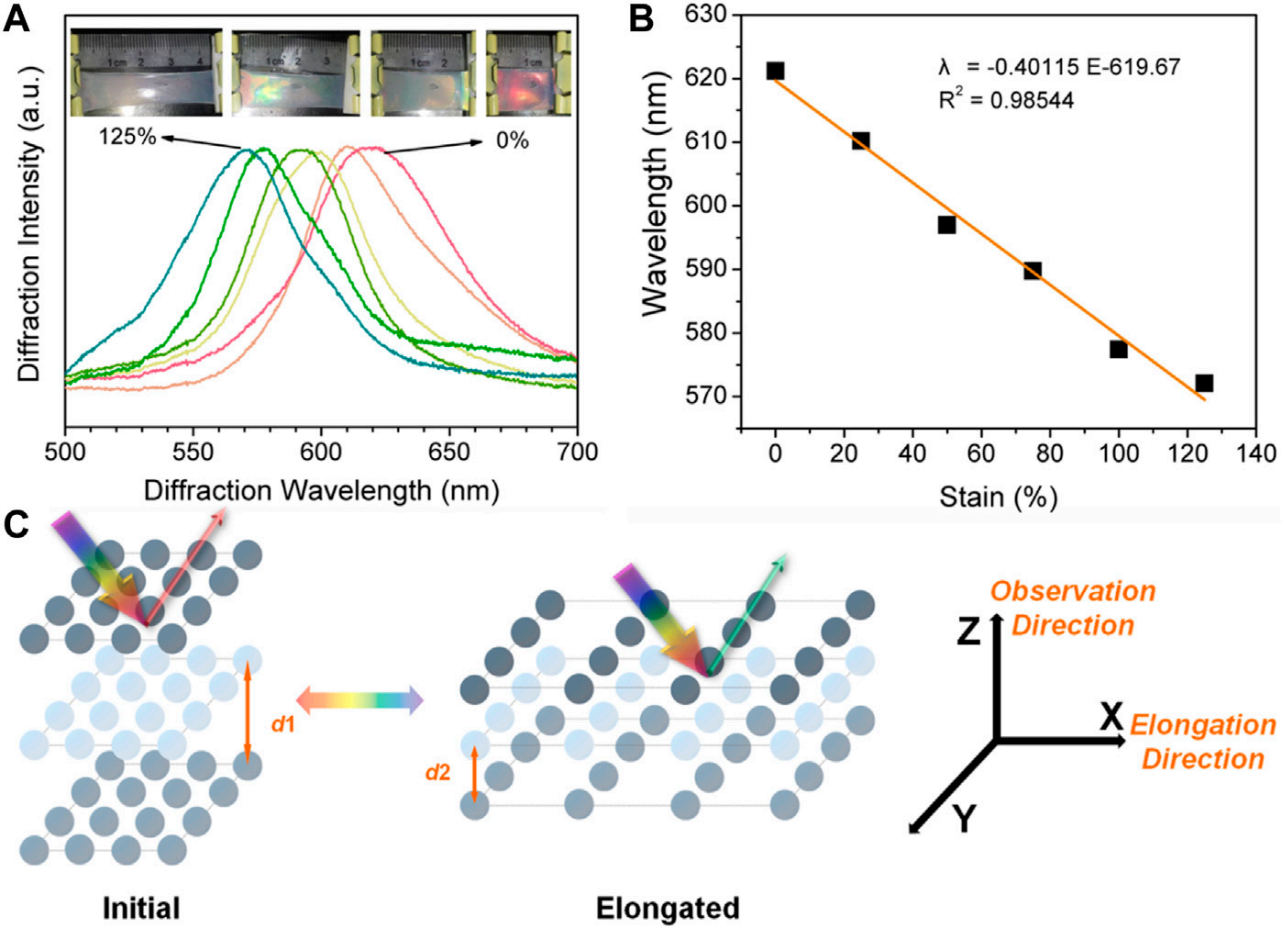
**(A)** The shift of diffraction wavelength of PVA/CPC film during stretching, inserts are the optical photographs during deformation **(B)** The relationship between elongation and diffraction peak shift of PVA/CPC film **(C)** Schematic diagram that explains the structural color change of PVA/CPC film during stretching.

As the natural light is propagated along the CPC lattice, the refractive index remains constant, and the observed diffracted wavelength is then related to the lattice parameter of the cubic unit cell, in the case of CPC, that is the nearest-neighbor distance of PS nanospheres. As can be seen in Figure 2, the linear blue-shift caused by the elongation could be attributed to the deformation that shortened the lattice distance along the X-direction, thus the observed wavelength from the Z-direction shifted accordingly. Such deformation is different from the three-dimensional swell/shrink of the hydrogels in liquids. Moreover, we found that as the elongation of the PVA/CPC film reached about 125%, the film became transparent and structural color of the film can barely be observed. It is speculated that the reason for this situation is that the increase of tensile strength caused nonlinear elongation of the film, which increased the disorder of the CPC array. The elongation at break of PVA/CPC film measured in the tensile process is ∼150%, which also proves that such film has strong mechanical strength (Azadi et al., 2020; Fang et al., 2020).

**FIGURE 3:**
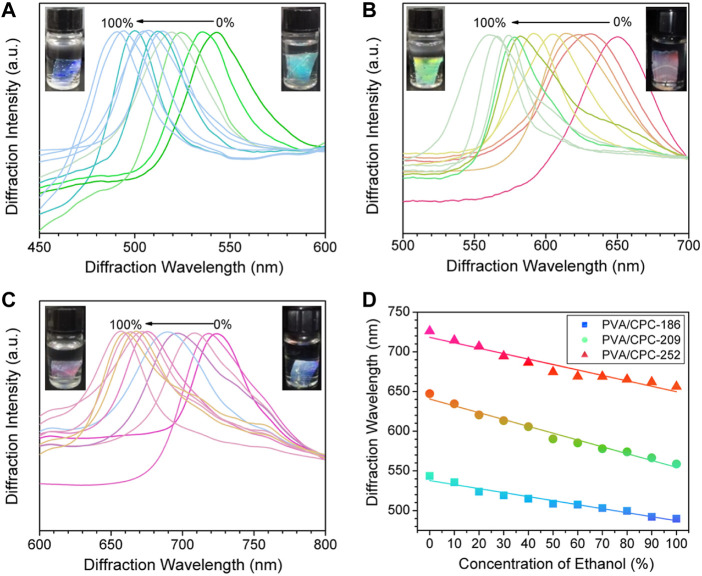
**(A–C)** The diffraction spectra of PVA/CPC-186, PVA/CPC-29 and PVA/CPC-252 films in different concentrations of ethanol aqueous solution, respectively; **(D)** Linear fitting between diffraction wavelength and ethanol concentration of PVA/CPC-186, PVA/CPC-209 and PVA/CPC-252 films, the color of each plot indicates the actual structural color changing during sensing process.

The ethanol sensing properties of PVA/CPC films were investigated. Samples prepared from PS nanospheres of 186 (±14, PDI = 0.21), 209 (±12, PDI = 0.23) and 252 (±18, PDI = 0.25) nm are noted PVA/CPC-186, PVA/CPC-209 and PVA/CPC-252, respectively, which were first fully swollen in water and then immersed in ethanol aqueous solution with different concentrations to reach swelling equilibrium. Figure 3 shows the diffraction wavelengths’ shifting of PVA/CPC films with the changing of the ethanol concentration. All the samples revealed noticeable continuous color change from pure water to pure ethanol. Specifically, PVA/CPC-186 showed bright cyan in pure water and turned blue in ethanol (Figure 3A), PVA/CPC-209 showed bright red in pure water and turned bright green in ethanol (Figure 3B), and PVA/CPC-252 showed purplish-red in pure water and turned reddish pink in ethanol (Figure 3C). Such blue-shift can be attributed to the shrinkage of the film, and the mechanism involving the change refractive index and the free energy of mixing.

The refractive index of ethanol is 1.362, which is slightly higher than that of water (1.333). Therefore, with the increase of ethanol concentration, the average refractive index of the solution increased accordingly. On the other hand, the equilibrium swelling degree of the PVA polymer film changed due to the change of free energy of mixing. According to Flory-Rehner theory (Flory and Rehner, 1943), during the swelling/shrink process, the osmotic pressure π of the polymer is the sum of three contributions:

$$\pi =\pi_{\text{mix}}+\pi_{\text{el}}+\pi_{\text{ion}}$$ (3)

where πmix is the force of polymer-solvent free energy of mixing, πel is the force caused by the deformation of polymer molecular chains to a more elongated state, and πion is the force from the nonuniform distribution of mobile counter-ions between the polymer and the solution media. For the non-ionic, free swelling condition, πion can be ignored, and πmix is taken as the pressure difference inside and outside the polymer film, which causes the swelling or shrinking of the film. As ethanol is a poor solvent for PVA, the equilibrium swelling degree of the film decreased with the increase of ethanol concentration, and the solvent transferred from the polymer film to the outside solution. The total change combined with the change free energy of mixing and the refractive index, as the decrease of equilibrium swelling degree is much greater than the increase of average refractive index, the increase of ethanol resulted in the blue-shift in diffraction wavelength of PVA/CPC film. Figure 3D plotted the diffraction shift of the composite films, for PVA/CPC-186, PVA/CPC-209, and PVA/CPC-252, the total shift was 52.5, 88.3, and 67.5 nm, respectively, which showed a good linear relationship with ethanol concentration. Thus the PVA/CPC films can be used as sensors for measuring ethanol content in aqueous solution with different color-shifting ranges as required.

**FIGURE 4:**
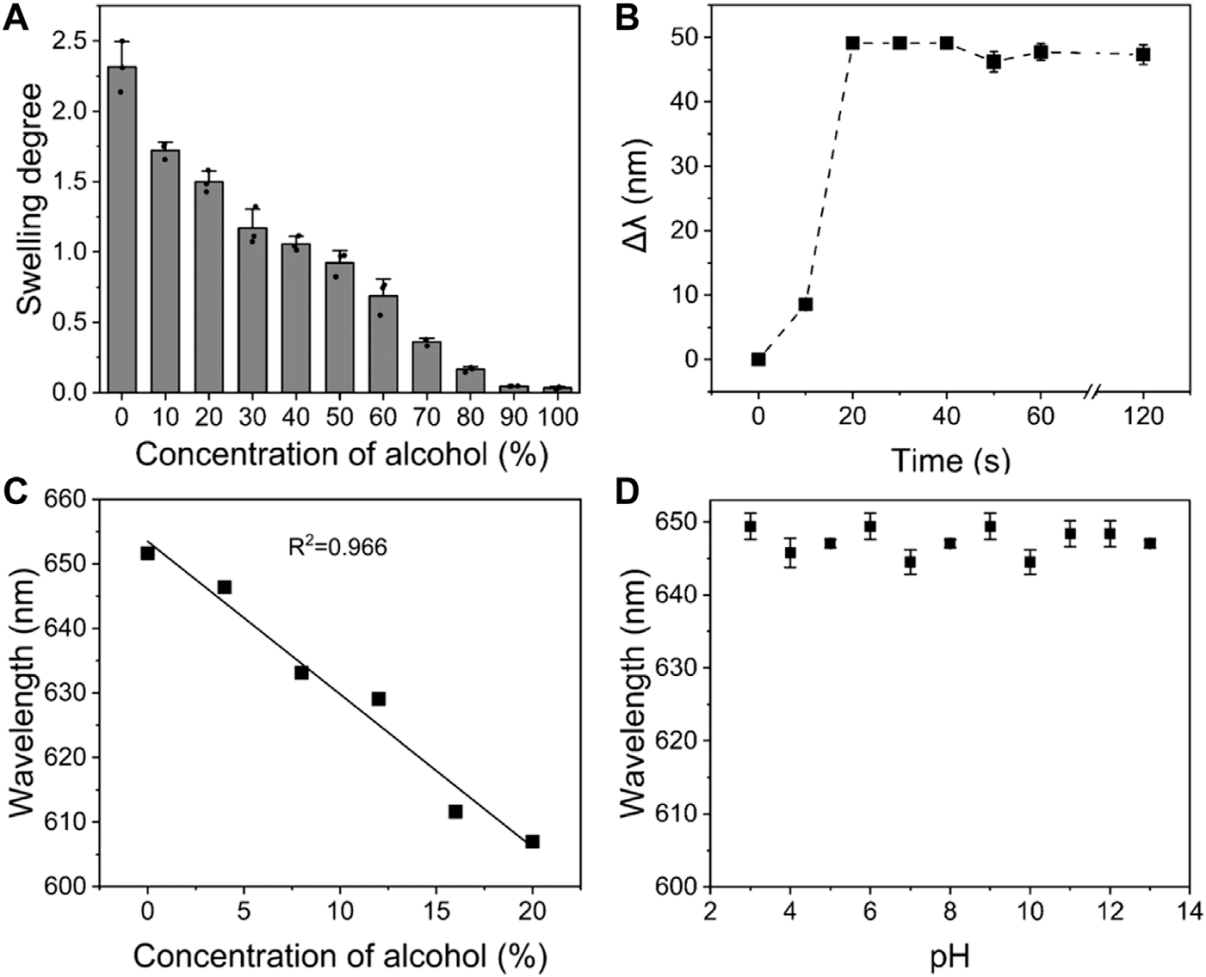
**(A)** Equilibrium swelling degree of PVA/CPC film in different concentrations of ethanol aqueous solution; **(B)** The relationship between diffraction wavelength shift and response time of PVA/CPC film in 20% ethanol aqueous solution; **(C)** The response of PVA/CPC film to low concentration ethanol aqueous solution within 20 s; **(D)** pH dependence of PVA/CPC film.

In addition, the accuracy, response time and sensitivity of PVA/CPC film to ethanol, which are essential parameters, were also tested. Firstly, the diffraction peak shifts at the concentration of 0–100% ethanol aqueous solution were tested repeatedly. It can be seen in Figure 4A that the PVA/CPC film had a stepped response to different ethanol concentrations, with good repeatability and tiny error. Then, the PVA/CPC film was immersed in a 20% ethanol aqueous solution and the diffraction spectrum of PVA/CPC film was captured every 10 s, and the average value was obtained by repeated measurement three times. Figure 4B shows the dynamic response curve of PVA/CPC film in 20% ethanol aqueous solution. It can be seen that the diffraction wavelength of PVA/CPC film rapidly blue-shifted with the extension of time. The diffraction wavelength red-shifted ∼49 nm within 20 s and remained unchanged after which. Therefore, the response time of PVA/CPC film can be considered no more than 20 s, which proves that the sensor film has a rapid response to ethanol, and such quick response might be attributed to the non-hydrogel state of the PVA polymer that easier to swell/deswell than hydrogels. Further, we investigated the PVA/CPC film for the detection at lower ethanol concentrations (4, 8, 12, 14, 16, and 20%) to verify the sensitivity, and the PVA/CPC film was immersed in the different solution to be tested for 20 s, and the diffraction wavelength was recorded.

As shown in Figure 4C, the diffraction wavelength of PVA/CPC film maintained a good linear relationship at lower ethanol concentrations with rapid response property, which can be used for more accurate detection of low concentration ethanol. Such quick response to low concentration ethanol may also be related to the properties of PVA bulk film. Moreover, the anti-interference property of the PVA/CPC film was also investigated in different pH media from 3 to 13. Figure 4D showed that the diffraction wavelength of PVA/CPC film affected by pH showed no more than 5 nm shift, the performance proved the stability of PVA/CPC film in various solutions.

**FIGURE 5:**
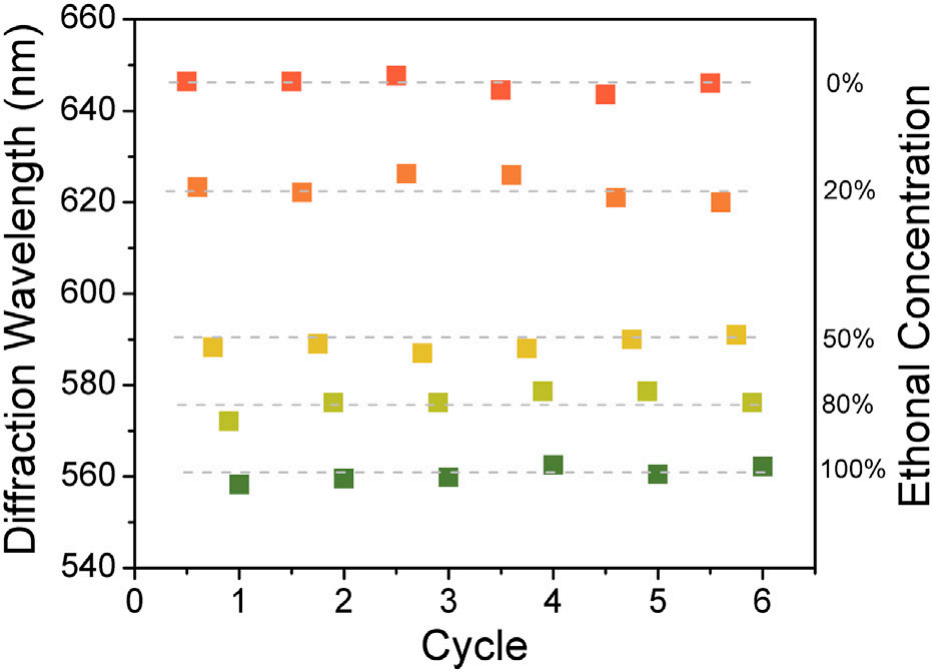
Repeatability test of PVA/CPC film response to different ethanol aqueous solutions.

Then, the reusability of sensors is investigated to evaluate the performance of the PVA/CPC sensor. The sensor film was alternately immersed in pure water and ethanol aqueous solutions with different concentrations (0, 20, 50, 80 and 100%) to detect whether the sensor could be reused. Figure 5 shows the diffraction peak position of PVA/CPC film in different sensing cycles. It is obvious that during six sensing cycles, the film showed identical diffraction wavelength to certain ethanol solution, showing good repeatability and reusability.

**FIGURE 6:**
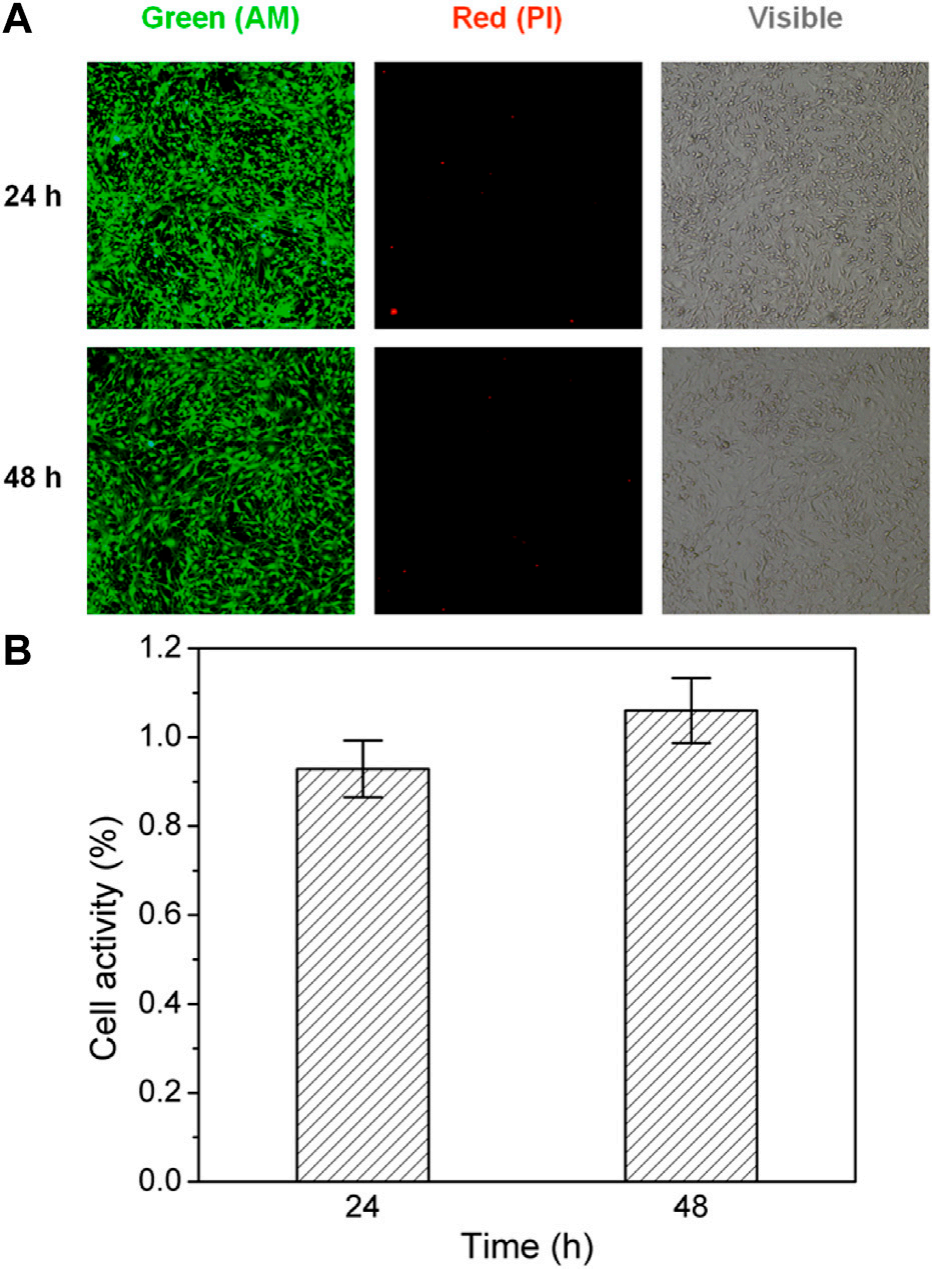
**(A)** IFM micrographs of cell-cultured PVA: Green (Calcein AM) and red (PI) fluorescence micrographs and visible morphology in 24 and 48 h; **(B)** Living cell statistical analysis showed good cellular activity (*p* > 0.05, *n* = 3).

Although the PVA/CPC film sensor was constructed with biocompatible PVA material, the biocompatibility was also examined by cell proliferation activity and fluorescence micrographs observation. As presented in Figure 6, both 24-h and 48-h IFM results showed similar cell density, and almost no increased dead cells were found with negative PI. This indicates the PVA/CPC films have good biocompatibility with biomedical potential in the future.

## Conclusion

A simple preparation method of non-hydrogel polymer-based photonic crystal film was presented. The film was prepared by combining biocompatible PVA and structural color reflecting colloidal photonic crystal structure. The difference in swelling properties of PVA in water and ethanol leads to the difference in equilibrium swelling degree. Thus the film can be utilized as an ethanol sensor, and the color change of which can directly be distinguished by the naked eye. The sensor showed a good linear relationship with diffraction wavelength and ethanol concentrations with rapid response within 20 s. It also proved with good accuracy (∼0.9 nm shift in diffraction wavelength for 1% alcohol concentration), repeatability (for at least six cycles), and stability (anti-interference in various pH).

## Data Availability

The original contributions presented in the study are included in the article/Supplementary Material, further inquiries can be directed to the corresponding authors.
