# Supplementary material for: Non-gelated polymeric photonic crystal films
Source: Front Chem. 2022 Sep 20;10:1009669. doi: 10.3389/fchem.2022.1009669 (PMC9531271; doi:10.3389/fchem.2022.1009669)
Supplement: Supplementary file 1 [file DataSheet1.pdf]

Non-Gelated Polymeric Photonic Crystal Film

Zhaoran Chu [1#], Zheng Ding [2#], Xuanjun Ning [1], Yimihan A [1], Menghan Wang [1], Kan Shao [3], Wenwei Tang [4], Cheng Chen [1,5] * and Jianzhong Bai [2] *

[1] School of Energy and Materials, Shanghai Polytechnic University, Shanghai 201209, China;

[2] Department of Orthopaedics, Tongren Hospital, Shanghai Jiao Tong University School of Medicine, Shanghai 200336, China;

[3] Department of Endocrinology, Tongren Hospital, Shanghai Jiao Tong University School of Medicine, Shanghai 200236, China;

[4] Modern Service Department, College of International Vocational Education, Shanghai Polytechnic University, Shanghai 201209, China;

[5] Shanghai Engineering Research Center of Advanced Thermal Functional Materials, Shanghai Polytechnic University, Shanghai 201209, China

* Correspondence: chencheng@sspu.edu.cn (C.C.) and bjz1851@shtrhospital.com (J.B.)

The morphology of PS nanospheres during polymerization was collected. After the addition of the initiator, a drop of the reaction mixture was collected at certain time and dried on a glass slide. With the increase of the reaction time, the particle size of nanospheres as shown in Figure S1, the diffraction color of the PS nanospheres showed bule, green and red as the reaction lasted for 5, 10 and 30 min. It is obvious that the particle size of PS nanospheres grew rapidly with the reaction time, and the structural colors is changed accordingly, thus the particle size of synthesized PS nanospheres can be controlled by adjusting the reaction time after initiating.

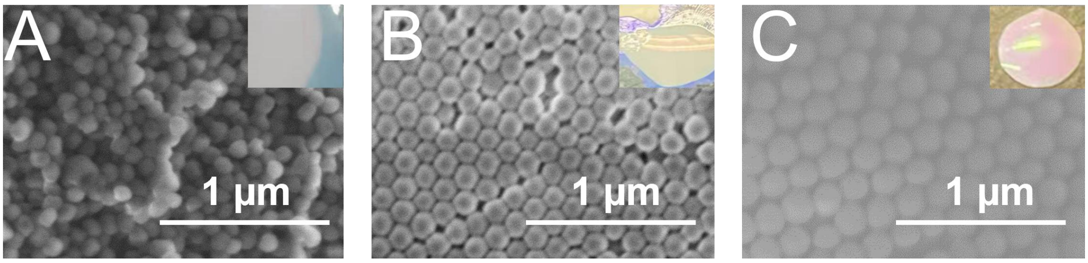

Figure S1. SEM images of PS nanospheres after adding initiator during polymerization: (A) 5 min; (B) 10min; (C) 30 min. The inserts are the corresponding photographs of the PS sample.

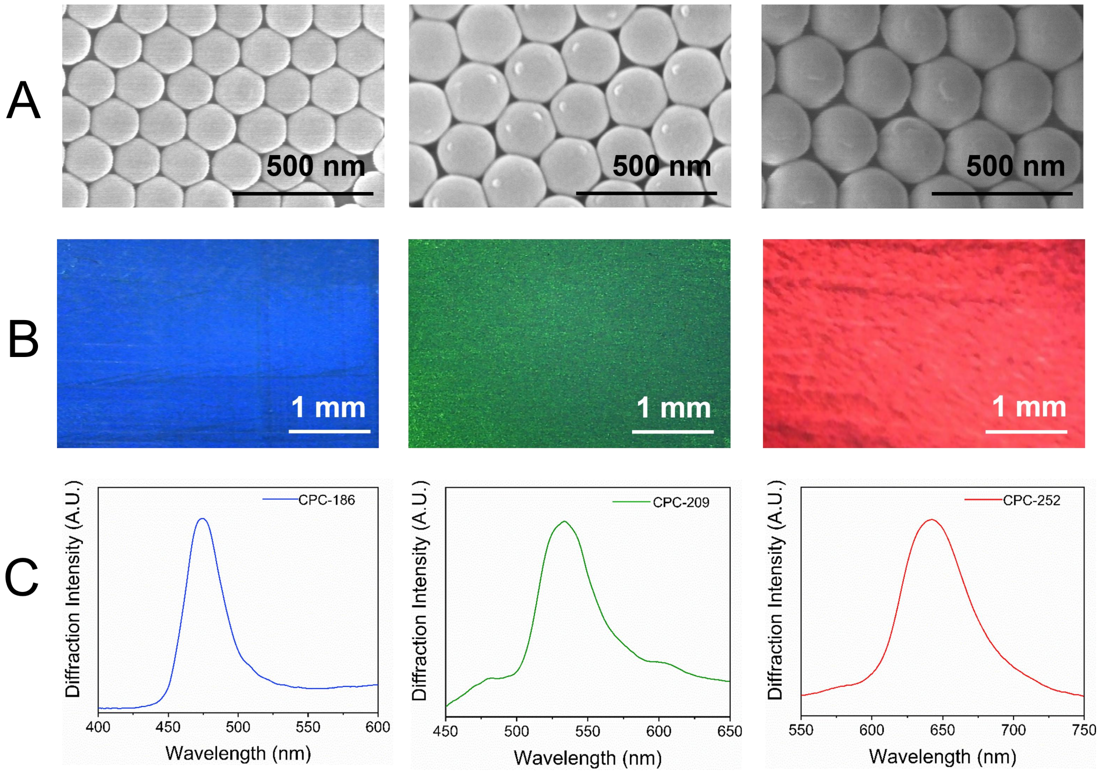

Figure S2. (A) SEM photographs of CPC samples; (B) Optical photographs showing the structural color of CPC samples; (C) Diffraction spectra of CPC samples. The particle sizes of PS nanospheres from left to right are 186, 209 and 252 nm, respectively.

In this paper, the synthesized PS nanospheres with three particle sizes (186, 209 and 252 nm) were prepared into CPC templates (CPC-186, CPC-209 and CPC-252) by vertical evaporation deposition method respectively. The scanning electron

microscope (SEM) images are shown in Figure S2A. It can be observed that the (111) surface of face –centered-cubic (FCC) CPC, and the CPC array is closely packed into an ordered periodic structure, and the particle size of PS nanospheres is monodispersed. Figure S2B shows the bright structural colors corresponding to CPC-186, CPC-209 and CPC-252, which are blue, green and red, respectively. As shown in Figure S2C, CPC sample has a sharp diffraction peak, indicating that the uniform CPC array is formed. The diffraction wavelengths of CPC-186, CPC-209 and CPC-252 are 472.72, 533.56 and 643.56 nm respectively.

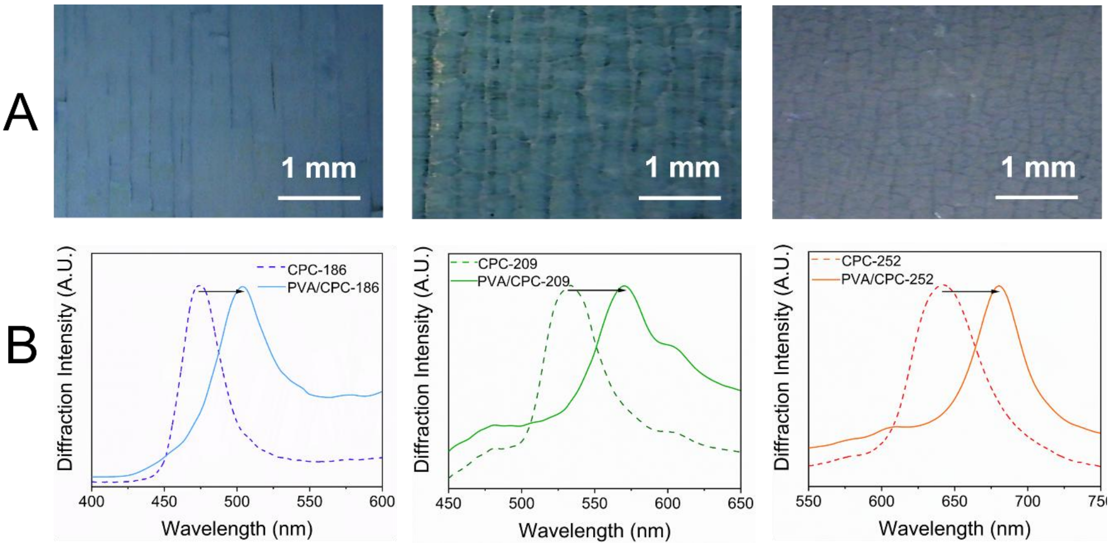

Figure S3. (A) Optical photographs of structural color of PVA/CPC films; (B) Diffraction spectra of PVA/CPC films. The particle sizes of PS nanospheres from left to right are 186, 209 and 252 nm, respectively.

Theoretically, the diffraction wavelength of CPCs should have linear relationship with the particle sizes of PS nanospheres, which follows the Bragg's law:

$$m\lambda_0 = 2n_a d_{111}\mathrm{Sin}\theta\,, \qquad \text{(S1)}$$

where m is the reflection series, λ_0 is the diffraction peak wavelength, n_a is the average refractive index, θ Is the angle between the incident light and the normal, and d_{111} is the lattice spacing.

Considering only the first-order diffracction, equation (S1) can be rewritten as:

$$\lambda_0 = 2n_a\sqrt{2/3}D\,, \qquad \text{(S2)}$$

where D is the diameter of PS nanospheres, and the average refractive index n_a can be expressed as:

$$n_a^2 = \sum_i n_i^2\varphi_i\,, \qquad \text{(S3)}$$

where φ Is the volume fraction of CPC template. Therefore, according to the above equations, the theoretical values of the CPC template constructed by PS nanospheres with different particle sizes and the reflection wavelength of PVA/CPC can be all calculated.

As PVA is completely filled into the air fraction of CPC to form PVA/CPC films, PS CPCs still have monotonous structural colors as shown in Figure S3A. It can be seen from Figure S3B, as PVA is filled into the gap of CPC, the diffraction peaks corresponding to PVA/CPC-186, PVA/CPC-209 and PVA/CPC-252 films red sifted to 503.76, 570.39 and 680.59 nm, respectively, due to the change of average refractive index.

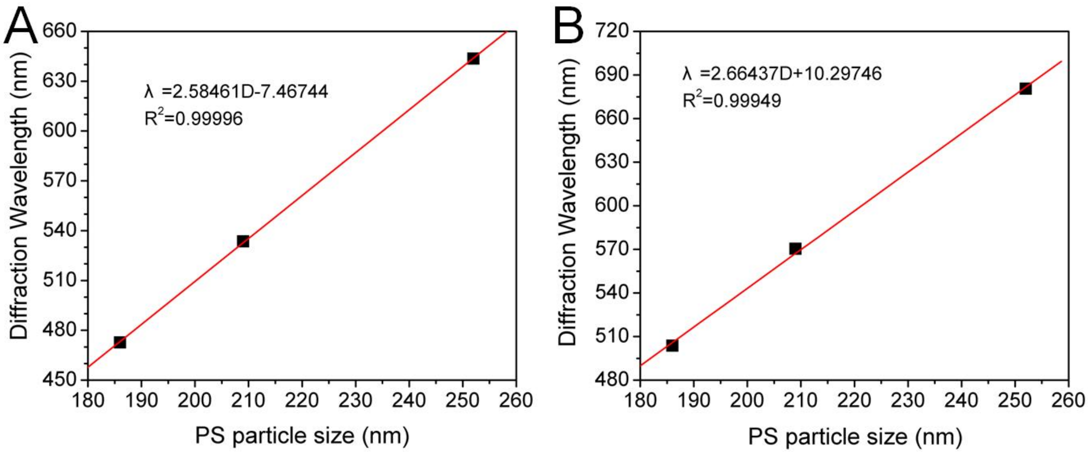

Figure S4. (A) The relationship between the diffraction wavelength of CPCs and the particle size of PS nanospheres; (B) Relationship between diffraction wavelength of PVA/CPC films and particle size of PS nanospheres.

Moreover, in Figure S4A, a good linearity was shown between the diffraction wavelength of CPC samples and the particle sizes of the PS nanospheres. According to Eqn (S2), as the lattice spacing of CPC remains unchanged, and thus a similar linearity was shown in Figure S4B, indicating good periodic structures were prepared.

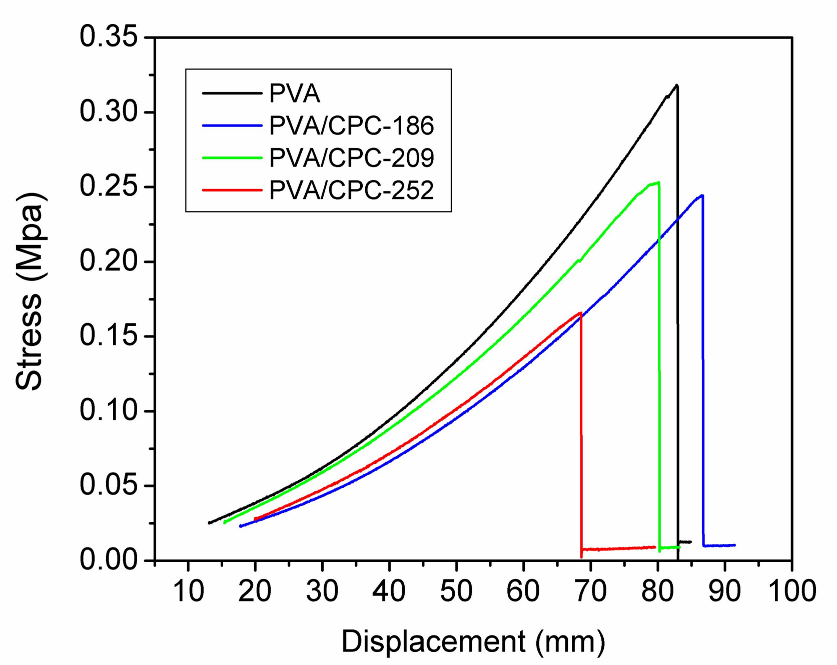

Figure S5. Stress-displacement curve of the PVA and PVA/CPC samples. The PVA/CPC combined sample showed comparable mechanical properties with pure PVA film.

Table S1 Sample parameters and results of tensile test

Sample	Width (mm)	Length (mm)	Thickness (mm)	Tensile displacement at maximum load (mm)	Tensile stress at maximum load (MPa)	Tensile stress at break (standard) (MPa)	Maximum load (N)
PVA	6.34	30	2.01	68.79818	0.24442	0.01044	3.11481
PVA/CPC-186	5.27	30	2.17	64.74849	0.25308	0.00895	2.89424
PVA/CPC-209	5.62	30	2.08	69.69823	0.31819	0.01264	3.71953
PVA/CPC-252	5.33	30	1.99	48.5985	0.16592	0.00903	1.75983

Tensile strength (TS, stress at fracture) was measured at room temperature using a Shimadzu AG22000A universal test machine at a crosshead speed of 100 mm min^{-1} according to GB/T 528-2009 standard.

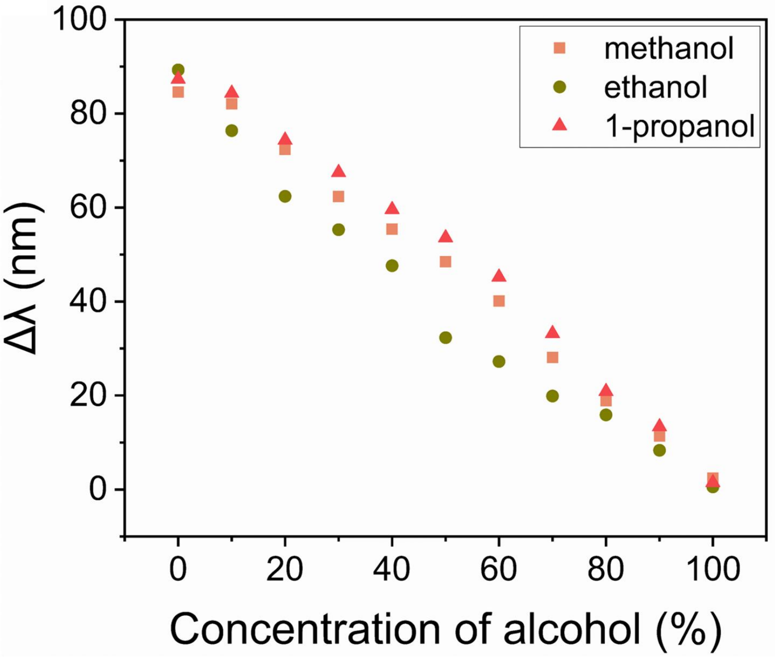

Figure S6. The response of PVA/CPC films to different concentrations of ethanol, methanol, and 1-propanol aqueous solution.

In order to explore the universality of PVA/CPC film sensor to other organic solutions, the responsiveness of PVA/CPC film to methanol and 1-propanol aqueous solutions was tested as comparison to ethanol. The PVA/CPC films were immersed in methanol and n-propanol aqueous solution with different concentrations, respectively, and the change of reflection wavelength (Δλ) was recorded. As shown in Figure S6, for all alcohol aqueous solution, with the increase of alcohol concentration, the reflection wavelengths of PVA/CPC films all showed blue-shift. Such universe response can be attributed to the change of free energy of mixing of PVA that showed similar low swelling capacity in organic solvent. Therefore, with the increase of

alcohol concentration, the swelling degree of PVA/CPC film decreases, resulting in the decrease of CPC lattice spacing, that is, the blue-shift of reflection wavelength. Although PVA/CPC film has no specific identification at present, we believe that this research provides a research idea, and the sensor film with specific identification characteristics can be further prepared based on the modification of this material.
